# A rare case of Goltz syndrome: Clinical presentation and diagnostic challenges

**DOI:** 10.1016/j.jdcr.2026.04.051

**Published:** 2026-05-02

**Authors:** Omar Nabil Turkistani, Renad A. Abbas, Saleh Mohammad Aldraibi, Raneem Majed Alqahtani, Abdulhadi Jfri

**Affiliations:** a College of Medicine, King Abdullah International Medical Research Center, King Saud bin Abdulaziz University for Health Sciences, Ministry of the National Guard, Saudi Arabia; b Department of Dermatology, King Abdullah Medical City, Jeddah, Saudi Arabia; c KAIMRC, King Abdullah International Medical Research Center, Jeddah, Saudi Arabia; d College of Medicine, King Saud bin Abdulaziz University for Health Sciences, Jeddah, Saudi Arabia; e King Abdullah International Medical Research Center, Jeddah, Saudi Arabia; f Division of Dermatology, King Abdulaziz Medical City, Jeddah, Saudi Arabia

**Keywords:** focal dermal hypoplasia, genodermatosis, Goltz syndrome, Gorlin-Goltz syndrome, X-linked disorders

## Case presentation

**Fig 1:**
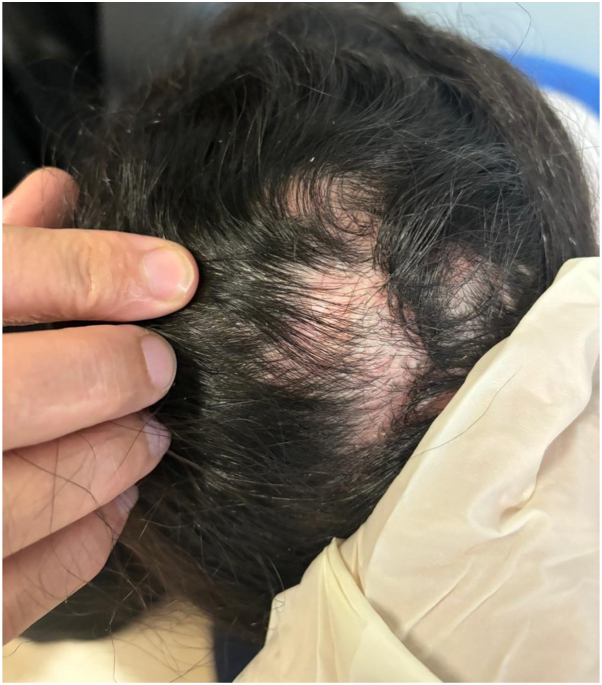
Scaly and erythematous plaques over the scalp.

**Fig 2:**
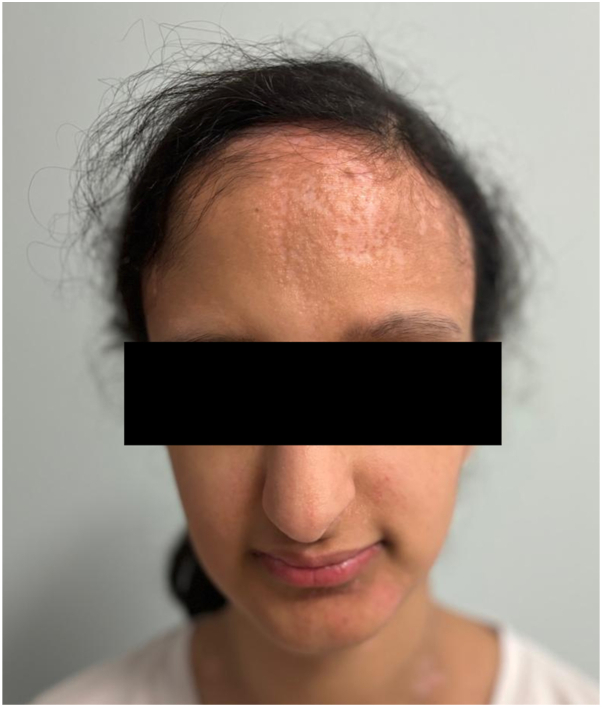
Mottled dyspigmentation over the forehead and a beaked nose.

**Fig 3:**
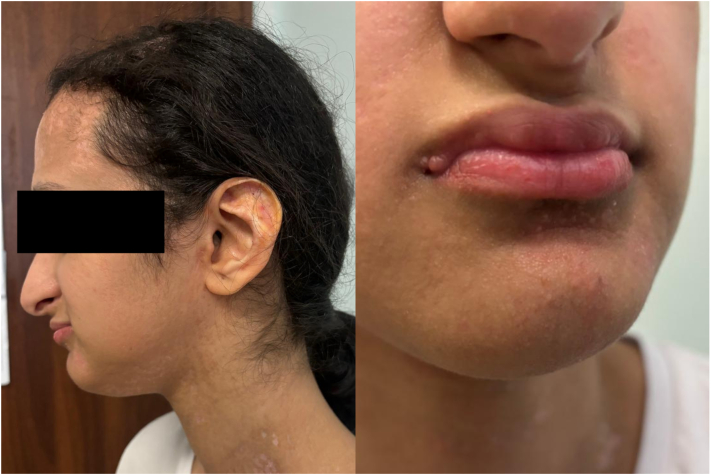
A small single oral papilloma at the right oral commissure, and large, back-slanted ears.

**Fig 4:**
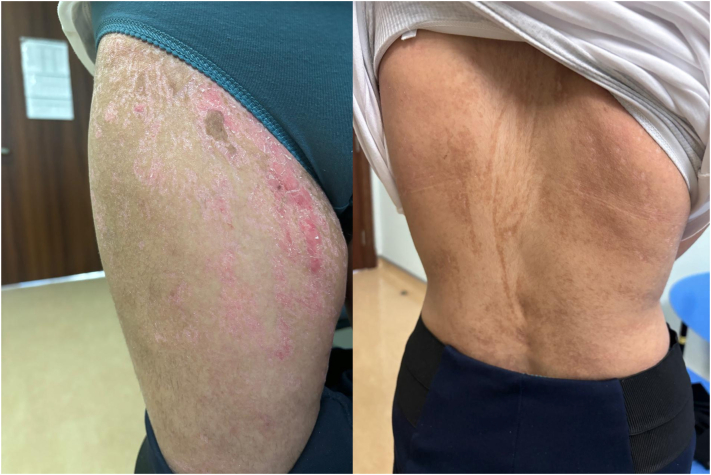
Linear Blaschkoid hyperpigmentation is seen on the back of the patient. Erythematous scaly plaque on the right thigh.

**Fig 5:**
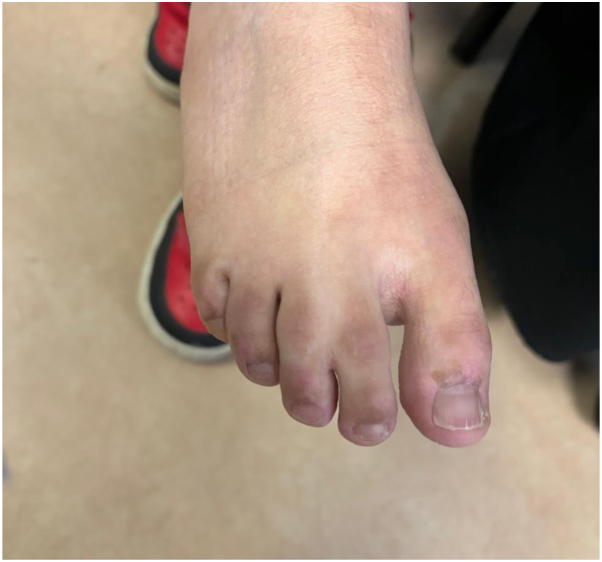
Nail dystrophy with onycholysis and V-nicking. Syndactyly of the toes on the right foot.

A 15-year-old female presented to the dermatology clinic with her mother, reporting multiple skin lesions. At birth, she was noted to have hypopigmented and hyperpigmented streaks on her back following the lines of Blaschko, which became more prominent during puberty. Developmental milestones and intellectual function were appropriate for her age. Family history was notable for parental consanguinity (first-degree relatives), maternal recurrent miscarriages, and paternal epilepsy. On examination, the patient was noticed to have erythematous scaly plaques over the scalp (Fig 1) , mottled dyspigmentation over the forehead and a beaked nose (Fig 2), a small single oral papilloma at the right oral commissure and large back-slanted ears (Fig 3), Blaschkoid hyperpigmentation in a linear pattern over multiple areas of the body with multiple erythematous scaly psoriasiform plaques over her arms and legs (Fig 4), multiple nail changes with onycholysis, V-nicking, and syndactyly in her right foot (Fig 5). Although no fat herniation was observed, the collection of clinical findings was highly suggestive of Goltz syndrome. To confirm the diagnosis by genetic testing, a trio-based whole exome sequencing analysis identified a heterozygous pathogenic de novo copy number variant encompassing axons 5, 6, and 7 of the *PORCN* gene. This finding confirmed the genetic diagnosis of X-linked dominant focal dermal hypoplasia. The management plan aimed for symptomatic relief of the skin rash by prescribing topical treatments, including ketoconazole 2% shampoo applied twice weekly for 15 weeks, mometasone furoate 0.1% lotion applied twice daily, petrolatum (Vaseline) ointment applied twice daily, betamethasone dipropionate 0.05% ointment applied twice daily, and regular use of moisturizing cream.

**Question 1: Which of the following features is MOST characteristic of Goltz syndrome (focal dermal hypoplasia)?**

**A.** Mutations in the IKBKG (NEMO) gene
**B.** Inflammatory vesicular and verrucous cutaneous stages in infancy
**C.** Structural dermal hypoplasia with skeletal abnormalities
**D.** Predominant involvement of ectodermal tissues only
**E.** Retinal vascular disease with neurologic sequelae

**Answer: C**

## Discussion

Goltz syndrome (focal dermal hypoplasia) should be suspected in female patients presenting with congenital Blaschkoid hypo- or hyperpigmented skin lesions, dermal hypoplasia, and mucocutaneous papillomas, particularly when associated with skeletal abnormalities such as syndactyly, nail dystrophy, and craniofacial dysmorphism, reflecting involvement of both ectodermal and mesodermal derivatives.^1^, ^2^, ^3^ The diagnosis is primarily clinical and is confirmed by identification of a pathogenic mutation or copy number variant in the *PORCN* gene, which plays a critical role in Wnt protein modification and secretion, thereby affecting multiple developmental pathways.^1^^,^^4^^,^^5^ This genetic finding distinguishes Goltz syndrome from other X-linked dermatoses, such as incontinentia pigmenti, which is characterized by inflammatory cutaneous stages and mutations in the *IKBKG/NEMO* gene.^2^^,^^3^ As no curative therapy currently exists for Goltz syndrome, management is primarily symptom-directed and supportive, targeting individual manifestations. Dermatologic interventions such as topical treatments for erosive or pruritic lesions and laser therapy for atrophic skin changes may improve symptoms, whereas structural abnormalities, including limb defects or mucosal papillomas, often require surgical or multidisciplinary management.^1^, ^2^, ^3^

## Clinical importance

This case highlights the importance of recognizing the clinical features of Goltz syndrome, particularly Blaschkoid dyspigmentation in combination with skeletal abnormalities and mucocutaneous papillomas. Early recognition can guide appropriate genetic testing and prompt multidisciplinary care involving dermatologists, geneticists, and other specialists, ensuring proper evaluation of associated systemic findings and appropriate genetic counseling for the patient and their family.

## Conflicts of Interest

None disclosed.
